# Neoadjuvant Chemotherapy Compared with Surgery for Oesophageal Carcinoma: A Retrospective Study and Missing Evidence

**DOI:** 10.7150/jca.81588

**Published:** 2023-02-05

**Authors:** Yan Zheng, Keting Li, Xianben Liu, Haibo Sun, Zongfei Wang, Guanghui Liang, Xinshuo Zhang, Jiachao Ji, Wenqun Xing

**Affiliations:** 1 Department of Thoracic Surgery, The Affiliated Cancer Hospital of Zhengzhou University, Henan Cancer Hospital, Zhengzhou, Henan 450008, P. R. China; 2 Department of Statistics, LinkDoc Technology Co., Ltd., Beijing, China

**Keywords:** oesophageal cancer, surgery, lymphadenectomy, neoadjuvant chemotherapy

## Abstract

***Background:*** Negative evidence for the use of neoadjuvant chemotherapy (NAC) to treat oesophageal squamous cell carcinoma (ESCC) has been reported in Western countries in the past century. However, in China, most ESCC patients underwent paclitaxel and platinum-based NAC without evidence from local RCTs. Empiricism or a lack of evidence does not necessarily mean that the evidence is negative. However, there was no way to compensate for the missing evidence. The only way to obtain evidence is by conducting a retrospective study using propensity score matching (PSM) to compare the effects of NAC and primary surgery on overall survival (OS) and disease-free survival (DFS) among ESCC patients in China, which is the country with the highest prevalence of ESCC patients.

***Methods:*** From January 1, 2015, to December 31, 2018, a total of 5443 patients with oesophageal cancer/oesophagogastric junction carcinoma who underwent oesophagectomy were retrospectively identified at Henan Cancer Hospital. After PSM, 826 patients were selected for the retrospective study and divided into the NAC and primary surgery groups. The median follow-up period was 54.08 months. Toxicity and tumour responses to NAC, intraoperative and postoperative outcomes, recurrence, DFS and OS were analysed.

***Results:*** The postoperative complication rates were not significantly different between the two groups. The 5-year DFS rates were 57.48% (95% CI, 52.05% to 62.53%) for the NAC group and 49.93% (95% CI, 44.56% to 55.05%) for the primary surgery group (P=0.0129). The 5-year OS rates were 62.95% (95% CI, 57.63% to 67.79%) for the NAC group and 56.29% (95% CI, 50.99% to 61.25%) for the primary surgery group (P=0.0397).

***Conclusion:*** Compared with primary surgery, NAC with paclitaxel and platinum-based chemotherapy and two-field extensive mediastinal lymphadenectomy might be associated with long-term survival benefits among ESCC patients.

## Introduction

Oesophagectomy remains the cornerstone of current therapy for oesophageal squamous cell carcinoma (ESCC), but there is significant regional variance. Fifty-three percent of ESCC cases are located in China 1. Similar to the significant differences in regional distribution, the standard treatment varies widely across different areas based on evidence provided by local randomized controlled trials (RCTs). Two multicentre trials 2,3 and 2 meta-analyses 4,5 in Western countries suggested that neoadjuvant chemotherapy (NAC) did not provide any survival benefits when used to treat ESCC. However, NAC is the standard treatment for resectable ESCC in Japan 6,7. Additionally, an increasing amount of evidence has indicated that there is no difference in survival benefit for patients who undergo neoadjuvant chemoradiotherapy (NACR) or NAC 8-10. Wang et al. 8 reported that the one-year overall survival (OS) was 87.1% (115 of 132) in the NACR group and 82.6% in the NAC group (109 of 132) (P=0.30). If there is no difference in survival between NAC and NACR, then the financial burden and side effects of NACR cannot be ignored, and the therapeutic status of NAC needs to be clarified.

In China, although NEOCRTEC5010 demonstrated the survival benefits of NACR when compared with surgery alone 11, most ESCC patients still receive NAC 12. There is virtually no change in the daily clinical practice after NEOCRTEC5010 was published. Is the lack of change due to the discretion of the surgeon, empiricism, or stubbornness? There is no evidence supporting the use of NAC in China; however, a lack of evidence is not necessarily equal to negative evidence. Currently, due to the development of MIE and complete lymph node dissection, surgical treatment and lymphadenectomy are no longer the focus of the RTOG trial 8911 (USA Intergroup 113), “At the time of esophagectomy, tissue from the lymph nodes was sampled 2.” We emphasized that the comparison for systemic treatment should be based on unique and complete surgical local control and standard lymphadenectomy. Another concern in conducting this study is that paclitaxel/platinum-based chemotherapy has become the main NAC regimen used in China 13,14, despite a lack of high-level evidence supporting this approach 15. There are many clinical practices that are performed without the support of high-level evidence. However, there was no way to compensate for the missing evidence. The power of big data and real-world studies cannot be neglected.

The advancement of immunotherapy is another reason to research the current topic. It remains unclear how the “standard” treatment can be combined with PD-1 if there is no supportive evidence for NAC, and it is unclear how to select the proper control group. Therefore, we performed this retrospective, single-centre study at a high-volume centre using propensity score matching (PSM). The aim of the current study was to compare NAC with primary surgery (two-field extensive mediastinal lymphadenectomy) using PSM.

## Material and Methods

### Study Design

This was a retrospective cohort study. The academic review board of Henan Cancer Hospital approved the protocol, and the Ethics Review Committee of Henan Cancer Hospital officially approved this study. The approval number is 2021-KY-0050-001. The research was retrospectively registered at ClinicalTrials.gov, NCT05569668. Informed consent for the use of deidentified data was obtained from Henan Cancer Hospital. The work has been reported in accordance with the STROCSS criteria 16. The protocol can be found at https://www.clinicaltrials.gov/ct2/show/NCT05569668.

### Setting and Data Collection

**Fig 1:**
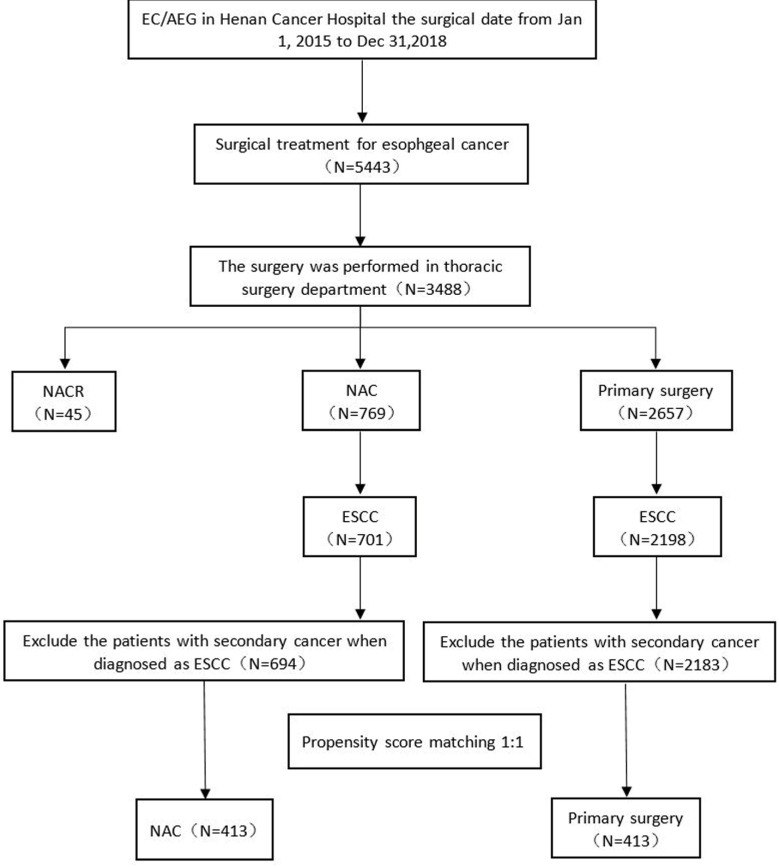
Patient distribution diagram. EC, oesophageal carcinoma; AEG, adenocarcinoma of the oesophagogastric junction; N, number; NACR, neoadjuvant chemoradiotherapy; NAC, neoadjuvant chemotherapy; ESCC, oesophageal squamous cell carcinoma.

The data were prospectively collected from the hubble of the LinkDoc data company at Henan Cancer Hospital between January 1, 2015, and December 31, 2018; patients who were diagnosed with oesophageal cancer/oesophagogastric junction carcinoma and underwent oesophagectomy were included. The inclusion criteria were as follows: (1) diagnosed with ESCC, (2) surgery performed in the thoracic surgery department, (3) no secondary carcinoma, and (4) no prior radiation or prior surgery. The patient files were retrieved by the data administrator. Patient treatment was determined by a local multidisciplinary team. A total of 5443 oesophageal cancer patients underwent surgery between January 1, 2015, and December 31, 2018. A total of 3488 oesophagectomy procedures were performed at the thoracic surgery department. Forty-five patients received NACR, 769 patients (including 701 ESCC patients) received NAC, and 2657 patients (including 2198 ESCC patients) underwent primary surgery. If we excluded patients with a second diagnosed carcinoma, there were 694 ESCC patients in the NAC group and 2183 ESCC patients in the primary surgery group (Fig. 1). The median follow-up was 54.08 months (interquartile range, 45.73-64.88 months). Data were analysed from July to October 2021. The simple deletion method was used to address missing data.

### Pretreatment Workup and Staging

The pretreatment staging examination included patient history, physical test, routine haematologic and biochemical tests, endoscopic ultrasound (EUS) and pathological examination, enhanced thoracic and upper abdominal computed tomography (CT) scanning, cervical and abdominal colour ultrasound, emission computed tomography (ECT), pulmonary function tests, and electrocardiography. Positron emission tomography (PET)/CT was also performed when patients were acceptable.

### Clinical and Pathological Tumour Effects

The clinical tumour responses were evaluated by the Response Evaluation Criteria in Solid Tumours 17. The clinical positive lymph nodes in radiographs were defined as the shortest diameter of lymph nodes > 0.8 cm and the ratio of long axis > 0.65. If there was no evidence of residual cancer cells, it was defined as a pathological complete response 18. The adverse events of NAC were evaluated by Common Terminology Criteria for Adverse Events (CTCAE) Version 5.0 19

### Treatment

Neoadjuvant Chemotherapy

Standard NAC comprised 2 cycles. Platinum plus paclitaxel or docetaxel was administered once every 3 weeks. Cisplatinum was administered at a total dose of 75 mg/m^2^ by continuous infusion on d1 or equally divided on Days 2-4 or Days 1-3. Paclitaxel was administered at a dose of 175 mg/m^2^ on d1 or at a dose of 87.5 mg/m^2^ on d1 and d8. If docetaxel was used, it was administered at a dose of 75 mg/m^2^ on Day 1.

### Surgical Procedure

At approximately 6-8 weeks after NAC, open (McKeown, left thoracic incision left cervical anastomosis) or MIE via thoracoscopy and/or laparoscopy was performed in the patients. Gastric tube reconstruction with a cervical anastomosis was performed to restore the continuity of the digestive tract. The range of lymphadenectomy included extensive mediastinal lymph node dissection. Bilateral laryngeal recurrent nerve lymph node dissection was requested for every patient. The abdominal nodes included the left gastric, para cardia, greater curvature, and lesser curvature. If the preoperative test showed that the resected neck lymph node had metastasized, then a 3-field lymph node dissection was needed. The main surgeon in our department had finished homogenized training for surgical techniques and processes. Each of them performed an average of approximately 80 oesophagectomies to treat cancer every year.

### Adjuvant Treatment

If positive lymph nodes were reported in the pathological test, then adjuvant chemotherapy was recommended. The chemotherapy regimen was the same as NAC. If the patients had R1-2 resection, adjuvant chemoradiotherapy was recommended.

### Follow-Up

One month after the operation, the patients were asked to visit the outpatient department. After that, the follow-up visit was every 3 months in the first 2 years, every 6 months in the third to fifth years and every year after 5 years until death. The LinkDoc company would also independently perform follow-up visits via phone. The data from follow-up visits were double checked. The routine visit examination included enhanced thoracic and upper abdominal computed tomography (CT) scanning and cervical and abdominal colour ultrasound. If the patients had a special complaint, other tests, such as oesophagogastroduodenoscopy and ECT, were added.

### Outcomes

Ninety days after surgery, morbidity and mortality were reported. The International Consensus on Standardization of Data Collection for Complications Associated with Oesophagectomy was used to evaluate postoperative complications 20. The Clavien-Dindo classification of surgical complications was applied to clarify the severity of the complications. OS was defined as the first day of patient admission to the inpatient department to the date of death from any cause. DFS was defined as the first day of patient admission to the inpatient department to the date of tumour recurrence confirmed by the follow-up tests.

### Statistical Analysis

**Fig 2:**
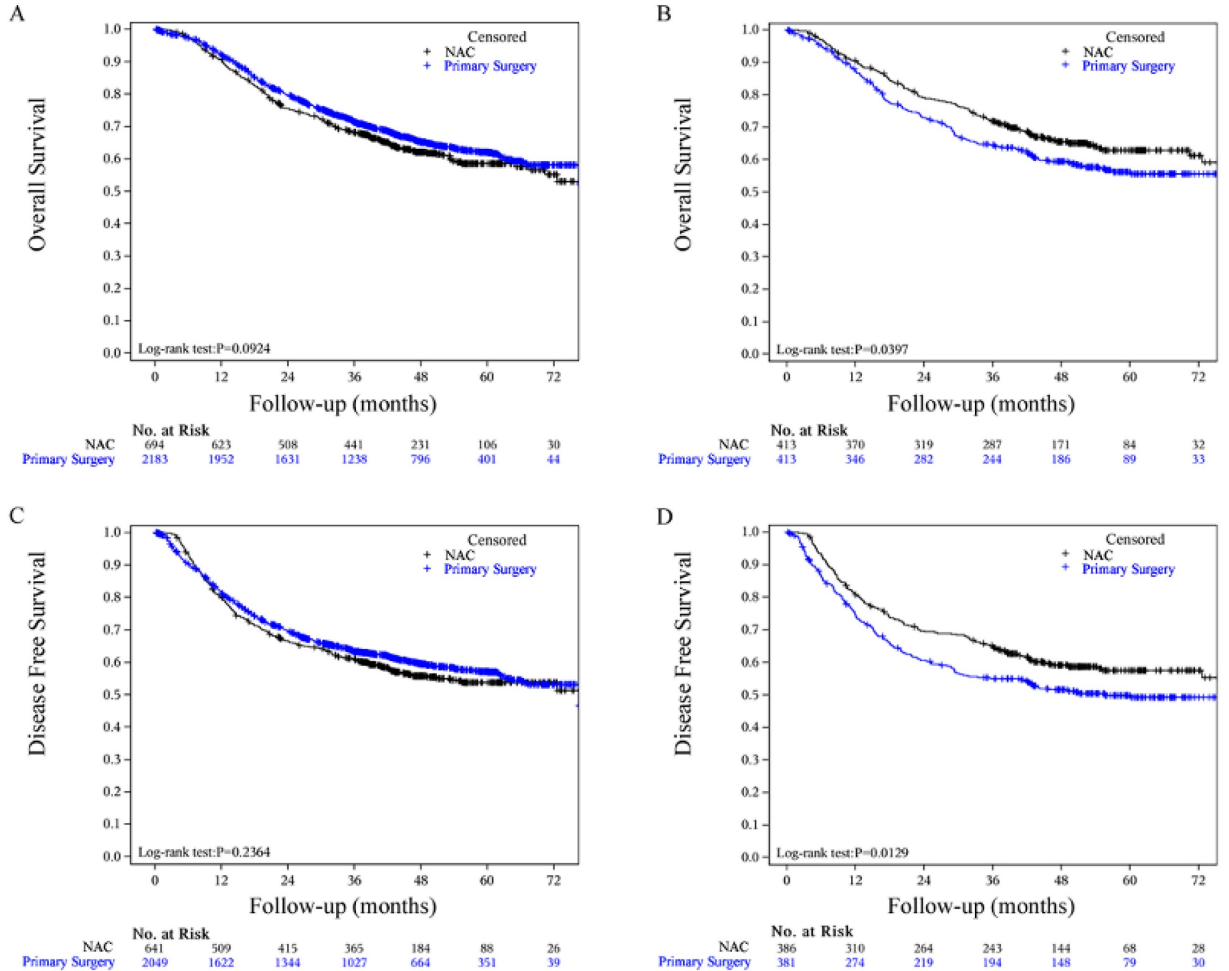
Kaplan‒Meier curves for the 5-year survival outcomes of propensity score matched patients and subgroup survival analysis. (A) Whole cohort OS, (B) Matched cohort OS, (C) Whole cohort DFS, (D) Matched cohort DFS. NAC, neoadjuvant chemotherapy.

**Fig 3:**
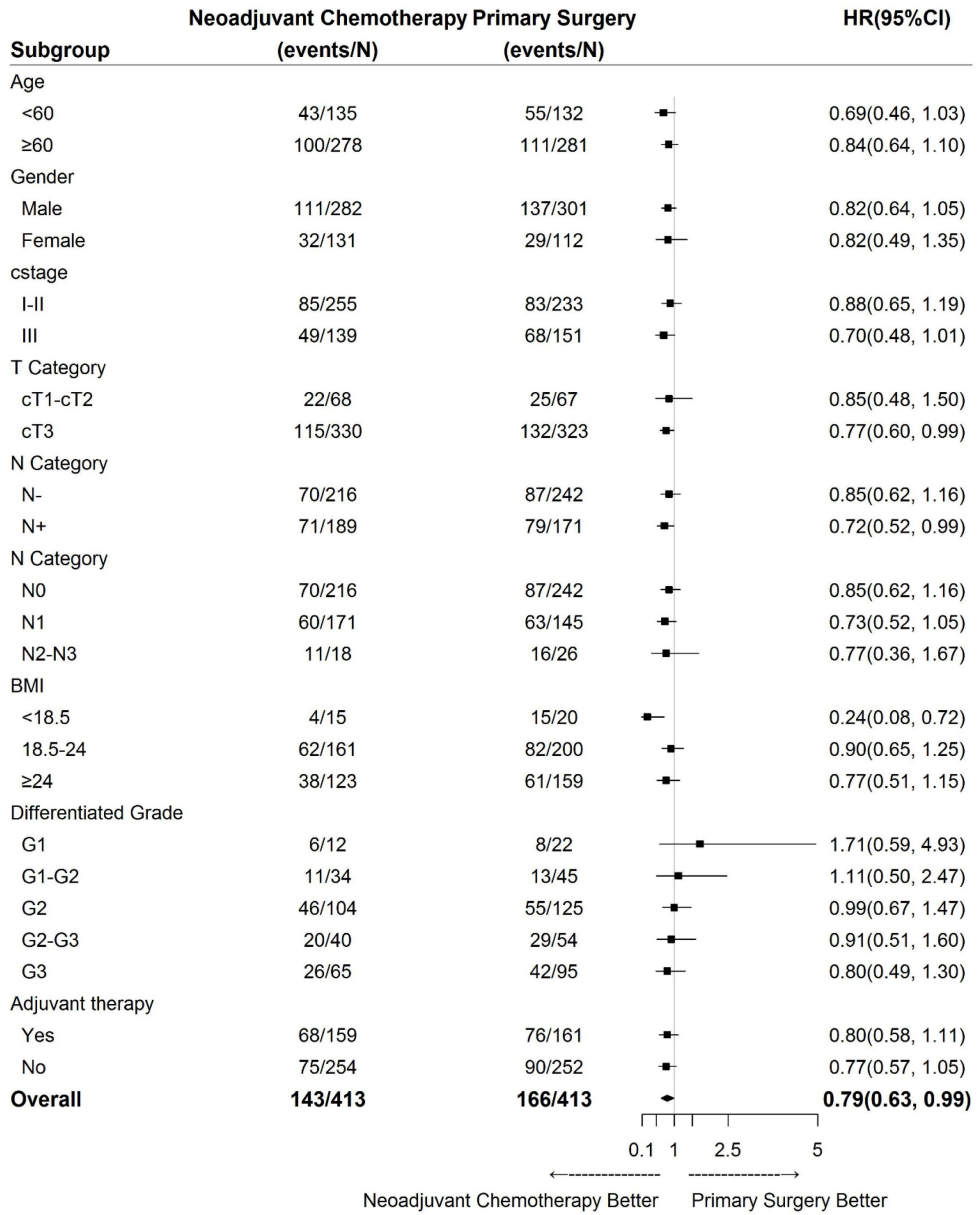
Heterogeneity of treatment effect based on the clinical data of ESCC. HR, hazard ratio; CI, confidence interval; cstage, clinical stage; cT, clinical tumour stage; N, lymph nodes; BMI, body mass index; G1, well differentiated; G2, moderately differentiated; G3, poorly differentiated.

Comparisons between the two groups in the clinicopathological variables were performed using the chi-square test, Mann-Whitney U test and Fisher's exact test for categorical parameters. The continuous variables were compared by the t test. Kaplan-Meier curves and a Cox proportional hazards regression model were adopted to perform OS analysis. R language 3.4.1 for Windows was used to complete the statistical calculations for all the tables. The OS and DFS are shown in Fig. 2. A p value<0.05 was defined as statistically significant. Subgroup analysis by NAC and primary surgery for OS in EC patients was explored (Fig. 3). To reduce bias, propensity score matching analysis was used. The variables, including age (<60 years, ≥60 years), sex, length of tumour (<4, 4-5, 5-6, 6-7, 7-8, 8-9, ≥9), clinical TNM stage, baseline differentiated degree, comorbidities, performance status, and nutritional status, were matched between the two groups. The statistical analysis was independently performed by statisticians of the LinkDoc company.

## Results

### Patients

A total of 5443 consecutive EC/AEG patients underwent surgical treatment between Jan 1, 2015, and Dec 31, 2018, in Henan Cancer Hospital (Fig. 1). Among them, a total of 3488 patients remained in the thoracic surgery department prior to surgery. A total of 769 patients received NAC, and 2657 patients underwent primary surgery. Patients with secondary cancer were excluded, and after PSM analysis, a total of 413 ESCC patients were matched. The characteristics of the full and PSM cohorts are summarized in Table 1. The primary data were comparable after PSM. No data were significantly different.

### Toxicities and Tumour Responses to Chemotherapy

A total of 694 full cohorts and 413 matched cohorts in the NAC group received neoadjuvant treatment. Adverse events during NAC are summarized in Table 2 based on CTCAE 5.0. A total of 92.94 (645) and 91.53% (378) of patients reported AEs in the full and matched cohorts, respectively. Most of these were grade II AEs (51.01% in the full cohort and 48.68% in the matched cohort). Leukopenia (4.70% in the full cohort and 5.21% in the matched cohort), thrombocytopenia (0.69% in the full cohort and 1.25% in the matched cohort) and neutropenia (14.17% in the full cohort and 14.19% in the matched cohort) had grade IV AEs. Pathological complete responses to NAC were observed in 25 (6.05%, 95% CI 4.1-8.0) patients in the full cohort and 12 (6.45%, 95% CI 4.5-8.4) in the matched cohort (Table 1).

### Intraoperative and Postoperative Outcomes

Surgical data are shown in Table 3. One patient in the NAC group and one patient in the primary surgery group underwent R2 resection. Four patients in the NAC group and 2 patients in the primary surgery group underwent exploratory operations. More patients in the NAC group received MIE (315, 76.64%), whereas more patients in the primary surgery group received open oesophagectomy (237, 58.81%). Except for severe thoracic adhesions, the selection of MIE and open surgery was not different. Some surgeons performed MIE only, and some surgeons performed open surgeries; these choices were based on the habit of the different surgeons. The number of harvested lymph nodes was higher in the primary surgery group (median, 19 vs. 22; mean, 22.1 vs. 22.8). There were no significant differences in other surgical data between the 2 groups (Table 3). There was also no significant difference in the complication rate between the two groups (Table 3).

### Progression-Free and Overall Survival

The median follow-up of the survivors in the PSM cohort was 51.61 months (95% CI, 50.37-53.49 months) in the NAC group and 55.76 months (95% CI, 54.31-58.06 months) in the primary surgery group. Twenty-nine (7.02%) patients in the NAC group and 35 (8.47%) patients in the primary surgery group were lost to follow-up; we were unable to contact them. Kaplan-Meier analysis for DFS and OS showed a significant difference between groups (Fig. 2). The two-sided log-rank P value of DFS was 0.0129. The DFS rates in the NAC group and primary S group were 57.48% (95% CI, 52.05% to 62.53%) and 49.93% (95% CI, 44.56% to 55.05%) at 5 years, respectively. The median DFS was not reached (Fig. 2D). A large difference between the two groups was observed in OS (log-rank test P = 0.0397). The OS rates in the NAC group and primary S group were 62.95% (95% CI, 57.63% to 67.79%) and 56.29% (95% CI, 50.99% to 61.25%) at 5 years, respectively. The median OS was not reached (Fig. 2B). There was no difference in OS (NAC group 58.61% vs. primary surgery group 62.11%) or DFS (NAC group 58.61% vs. primary surgery group 62.11%) at 5 years in the whole cohort. The survival curves for OS and DFS are shown in Fig. 2A and C, respectively. The local recurrence rate (p=0.2216) and distant metastasis (p=0.2078) were lower in the NAC group; however, these differences were not statistically significant (Table 3).

### Subgroup Analysis

The results of subgroup analyses of OS regarding age, sex, clinical stage, clinical lymph node status, clinical tumour depth, body mass index (BMI), differentiation grade and adjuvant therapy are shown in Fig. 3. Treatment was more effective in the NAC group among cT3 patients, cN+ cases (especially N1 cases), and patients with a BMI less than 18.5 (Fig. 3).

## Discussion

This retrospective study showed that, compared with primary surgery, NAC followed by surgery (two-field extensive mediastinal lymphadenectomy) significantly increased OS and DFS in patients with locally advanced ESCC. The risk of disease recurrence was 7.55% lower in the NAC group (HR, 0.76; 95% CI, 0.62 to 0.94), and the risk of death was 6.66% lower (HR, 0.79; 95% CI, 0.63 to 0.99). An NAC regimen (Taxol/paclitaxel and platinum-based chemotherapy) was the most clinically applied regimen in China, despite a lack of high-level clinical evidence. In this large cohort study, the regimen was shown to be safe and effective. This conclusion was the same as that of the Japan Clinical Oncology Group trial 9907 (JCOG9907) 21 but different from that of the RTOG trial 8911 (USA Intergroup 113) 2. The 5-year OS subgroup analysis showed that cN+ and cT3 patients gained more survival benefits from NAC. Compared with the survival data of the whole cohort, patients who received NAC in the matched cohort showed significant survival benefits (Fig. 2).

A reason for the positive conclusion might be the chemo-regimen. All these past negative trials were performed on chemotherapy regimens with 5-Fu in the last 2 decades 5. Paclitaxel/docetaxel showed a promising response rate for squamous cell carcinoma 22. It was soon adopted for ESCC. Triplicate regimens have also been developed. After the completion of Docetaxel+cisplatin+fluorouracil (DCF), the pathological complete response rate was 17% in a multicentre phase II clinical trial in Japan 23. The incidence of high-grade toxicity was shown in the DCF regimen. Grade III/IV neutropenia and febrile neutropenia (FN) were 56% and 20%, respectively 24. Currently, in China, the domain regimen for NAC of ESCC is paclitaxel and cisplatin (TP)/docetaxel and cisplatin (DP), without high-level evidence/RCTs. In a recent study, there were 28.32% and 27.29% Grade III/IV AEs in the matched and full NAC cohorts, respectively. The most concerning grade III/IV neutropenia was only 31.76% and 30.71% in the matched and full NAC cohorts, respectively, much less than previous triplet regimen reports 24.

The surgical data in the two groups showed some significant differences. More patients in the NAC group received MIE (76.64%), and more patients in the surgery group underwent open surgery (58.81%), p<0.001. The TIME trial showed no difference in the 3-year survival of patients who received MIE or the open approach 25. A meta-analysis of 17 separate case-control reports also reached the same conclusion 26. This difference may not affect OS in the two groups. The NAC group had a longer surgical time and shorter PODs, which may also be affected by the greater proportion of patients who received MIE in the NAC group. The NAC group had few harvested lymph nodes, which was consistent with previous studies and theories 27. The local recurrence (9.69% versus 12.35%, p=0.2216) and distant metastasis rates (8.96% versus 11.62%, p=0.2078) were lower in the NAC group; however, the difference was not statistically significant.

The conclusion of our study was different from that of many RCTs, such as the 8911 trial 2, and our own meta-analysis 5. Except for the dominant chemo regime. The surgical techniques were quite different from those in the last century. As the only local control method in NAC combination, surgical QC should be stricter 28. In the 8911 trial, there was no MIE, and the surgical experience was limited compared to that currently 2. There were 123 centres that joined the 8911 trial, and the recruitment lasted more than 5 years. As a low-incidence disease in Western countries, the experience might be limited. Third, different regions were used for lymphadenectomy. ESCC had early lymph node metastasis, and even T2 patients had laryngeal recurrent nerve lymph node metastasis. In our study, the bilateral laryngeal recurrent nerve lymph node must be removed, which has a 20.7% metastatic rate 29. However, the sampling of lymph nodes was acceptable at that time. The omission of positive lymph nodes might transform the surgery into an R2 resection, which might contribute to negative final results. Fourth, the R0 resection rates of primary surgery in 8911 2 and CROSS 30, 9907 21 were 59%, 69%, and 89%, respectively. With the development of CT/PET CT/MRI, the R0 resection rate was 91.2% in 5010 11. NAC should be more helpful for patients expected to undergo R0 resection, whereas NACR had OS benefits by increasing the R0 receipt rate in the CROSS trial.

This study has several limitations. First, this was a retrospective study. Some symptoms that were not digitized might generate selection bias. After PSM, more MIEs were performed in the NAC group, which might have led to bias. Second, the LinkDoc company was invited to independently manage the clinical database of the thoracic surgery department starting in 2015. Data collected before 2015, which had longer follow-up durations, were unavailable. Although the last follow-up date was 30 June 2021, longer follow-up results are needed. Third, the details of postoperative complications regarding the Clavien-Dindo classification have not been well established and are still missing data. Fourth, this study was conducted in the area with the highest proportion of ESCC in the world and in a high-volume cancer centre, so further investigation is needed to determine whether these results can be generalized to low-incidence regions.

## Conclusions

Our study suggested that the NAC TP/DP regimen might be safe and lead to better long-term survival outcomes than primary surgery. In the absence of evidence from RCTs in China, the current study provided the strongest evidence for TP/DP regimen NAC to date. We believe that the findings in this study are important to compensate for missing evidence associated with the commonly used NAC TP/DP in China and are also important for the choice of combined treatment with PD-1.

## Figures and Tables

**Table 1:** Characteristics of full and propensity score-matched cohorts

Characteristics	Full Cohort			Propensity Score Matched		
	NAC (n=694)	Surgery (n=2183)	P value	NAC (n=413)	Surgery (n=413)	P value
Gender			0.0034*			0.1468
Male	503(72.48)	1452(66.51)		282(68.28)	301(72.88)	
Female	191(27.52)	731(33.49)		131(31.72)	112(27.12)	
Age (years)			<0.001*			0.8234
<60	260(37.46)	623(28.54)		135(32.69)	132(31.96)	
>=60	434(62.54)	1560(71.46)		278(67.31)	281(68.04)	
Age median (range)	62(36-84)	64(29-86)	<0.001*	63(40-84)	64(40-82)	0.1178
Tumour length (cm) median (range)	6(1-19)	4(0.5-17)	<0.001*	5(1-19)	5(1-17)	0.8976
BMI median (range)	23.1(15.6-33.9)	23.10(14.3-35.0)	0.9888	23.10(15.6-33.6)	23.30(15.6-30.9)	0.6943
NA	183	170		114	34	
<18.5	25(4.89)	111(5.56)	0.7592	15(5.02)	20(5.28)	0.9584
18.5-24	288(56.36)	1097(54.90)		161(53.85)	200(52.77)	
≥24	198(38.75)	790(39.54)		123(41.14)	159(41.95)	
Smoking			0.0885			0.1674
NA	167	66		102	13	
Never	259(49.15)	1153(54.46)		169(54.34)	189(47.25)	
Ever	66(12.52)	231(10.91)		40(12.86)	57(14.25)	
Current	202(38.33)	733(34.62)		102(32.80)	154(38.50)	
Alcohol			0.0568			0.2620
NA	180	76		111	16	
Never	278(54.09)	1238(58.76)		176(58.28)	218(54.91)	
Ever	21(4.09)	55(2.61)		15(4.97)	13(3.27)	
Current	215(41.83)	814(38.63)		111(36.75)	166(41.81)	
Clinical TNM staging 7th ed N (%)			<0.001*			0.2960
NA	63()	135()		0	0	
Stage I	7(1.11)	193(9.42)		3(0.73)	3(0.73)	
Stage II	303(47.94)	1341(65.48)		252(61.02)	230(55.69)	
Stage III	321(50.79)	514(25.10)		158(38.26)	180(43.58)	
Weight (kg) median (range)	63(38-98)	62(34-110)	0.0797	63(40-96)	63(38-59)	0.8791
NA	183	170		114	188	
Histological Grade			<0.001*			0.7843
NA	11	47		158	22	
G1	23(3.37)	140(6.55)		12(4.71)	22(6.45)	
G1-G2	60(8.78)	318(14.89)		34(13.33)	45(13.20)	
G2	180(26.35)	666(31.18)		104(40.78)	125(36.66)	
G2-G3	75(10.98)	325(15.22)		40(15.69)	54(15.84)	
G3	345(50.51)	687(32.16)		65(25.49)	95(27.86)	
CMI positive N (%)	2(0.58)	7(0.72)	1.0000	2(0.91)	2(1.14)	1.0000
pCR N (%)	12(6.45)	NA	NA	25(6.05)	NA	NA
ypTNM stage						
I	68(9.80)	406(18.60)		39(9.44)	63(15.25)	
II	246(35.45)	868(39.76)		161(38.98)	144(34.87)	
III	292(42.07)	692(31.70)		172(41.65)	148(35.84)	
IVA	78(11.24)	191(8.75)		32(7.75)	51(12.35)	
IVB	10(1.44)	26(1.91)		9(2.18)	7(1.69)	
Cycles of NAC median (Q1, Q3)	2(1,2)	NA	NA	2(1,2)	NA	NA
Cycles of NAC			NA			NA
1~2	653(94.09)	NA		387(93.70)	NA	
3-4	40(5.76)	NA		25(6.05)	NA	
5	1(0.14)	NA		1(0.24)	NA	
Cycles of AC median (Q1, Q3)	2(1,2)	NA	NA	2(1,4)	NA	NA
AC N (%)			0.001*			0.886
Yes	287(41.35)	748(34.26)		159(38.50)	161(38.98)	
No	407(58.65)	1435(65.74)		254(61.50)	252(61.02)	

Abbreviations: NAC, neoadjuvant chemotherapy; N, number; BMI, body mass index; NA, not available; TNM, tumour/node/metastasis; G1, well differentiated; G2, moderately differentiated; G3, poorly differentiated; CMI, circumferential margin involvement; ypTNM, pathological tumour/node/metastasis; Q, quarter; AC, adjuvant therapy; * Statistically significant (*p*<0.05).

**Table 2:** Side effects of neoadjuvant therapy in the matched cohort

Toxicity	Matched NAC (n=413)	Full NAC cohort (n=694)
AE N (%)		
Yes	378(91.53) 35(8.47)	645(92.94)
No	35(8.47)	49(7.06)
CTCAE grade N (%)		
missing	35	49
I	87(23.02)	140(21.71)
II	184(48.68)	329(51.01)
III	81(21.43)	135(20.93)
IV	26(6.88)	41(6.36)
Leukopenia		
0	221	319
I	94(48.96)	166(52.04)
II	61(31.77)	96(30.09)
III	27(14.06)	42(13.17)
IV	10(5.21)	15(4.70)
Anaemia		
0	135	208
I	199(71.58)	344(70.78)
II	74(26.62)	133(27.37)
III	5(1.80)	9(1.85)
IV	0(0.00)	0(0.00)
Thrombocytopenia		
0	80	550
I	53(66.25)	101(70.14)
II	22(27.50)	37(25.69)
III	4(5.00)	5(3.47)
IV	1(1.25)	1(0.69)
Neutropenia		
0	265	440
I	52(35.14)	94(37.01)
II	49(33.11)	82(32.28)
III	26(17.57)	42(16.54)
IV	21(14.19)	36(14.17)
Total bilirubin increased		
0	125	486
I	95(76.00)	153(73.56)
II	29(23.20)	53(25.48)
III	1(0.80)	2(0.96)
IV	0(0.00)	0(0.00)
Creatinine		
0	395	661
I	17(94.44)	30(90.91)
II	1(5.56)	2(6.06)
III	0(0.00)	1(3.03)
IV	0(0.00)	0(0.00)
Emesis		
Yes	23(5.57)	51(7.35)
No	390(94.43)	643(92.65)
Diarrhoea		
Yes	2(0.48)	2(0.29)
No	411(99.52)	692(99.71)

NAC, neoadjuvant chemotherapy; N, number; AE, adverse effect; CTCAE, Common Terminology Criteria for Adverse Events.

**Table 3:** Intraoperative and postoperative outcomes of the two groups after PSM

Variables	NAC (N=413)	Surgery (N=413)	P value
Operation, N%			0.843
Radical resection	408	410	
Palliative resection	1(0.26)	1(0.26)	
Explora	4(1.02)	2(0.52)	
Surgical approach N%			0.0000*
Missing	2	10	
Open	96(23.36)	237(58.81)	
MIE	315(76.64)	163(40.45)	
Hybrid operation	0(0.00)	3(0.74)	
Open approach, N%			0.2145
Missing	96	237	
Left Thoracic Approach	41(42.71)	119(50.21)	
Right Thoracic Approach	55(57.29)	118(49.79)	
Anastomotic methods, N%			0.3390
Missing	0	4	
Intrathoracic anastomosis	3(0.73)	6(1.47)	
Cervical anastomosis	410(99.27)	403(98.53)	
Operative time (min)			<0.0001*
Mean (standard deviation)	283.2(79.46)	259.2(80.99)	
Median (Q1,Q3)	270.0(240.0, 330.0)	245.0(200.0, 310.0)	
Second operation, N%			1.0000
Yes	1(0.24)	1(0.24)	
No	412(99.76)	412(99.76)	
Blood loss (ml)			0.1371
Missing	90(323)	152(261)	
Mean (standard deviation)	179.0(118.72)	197.7(120.22)	
Median (Q1,Q3)	150.0(100.0, 200.0)	200.0(100.0, 200.0)	
Postoperative hospital stay			<0.0001*
Mean (standard deviation)	16.1(11.96)	17.4(9.91)	
Median (Q1,Q3)	13.0(9.0, 18.0)	14.0(13.0, 18.0)	
Maximum, Minimum	1, 113	4, 75	
Fasting days			0.2060
Mean (standard deviation)	8.8(13.03)	9.1(8.29)	
Median (Q1,Q3)	7.0(1.5, 9.5)	8.0(3.0, 11.0)	
Maximum, Minimum	1, 84	1, 42	
Chest tube drainage days			0.2687
Mean (standard deviation)	6.4(3.89)	7.4(3.66)	
Median (Q1,Q3)	6.0(3.5, 9.0)	7.0(5.5, 9.0)	
Maximum, Minimum	1, 13	1, 27	
Mediastinal tube drainage days			0.8690
Mean (standard deviation)	11.6(11.03)	8.7(4.30)	
Median (Q1,Q3)	8.0(6.0, 12.0)	6.0(6.0, 13.0)	
Maximum, Minimum	4, 42	3, 15	
Lymph nodes retrieved			0.0295*
Mean (standard deviation)	22.1(13.59)	22.8(10.97)	
Median (Q1,Q3)	19.0(12.0, 30.0)	22.0(15.0, 29.0)	
Positive lymph nodes			0.5008
Mean (standard deviation)	1.0(2.04)	1.3(2.45)	
Median (Q1,Q3)	0.0(0.0, 1.0)	0.0(0.0, 2.0)	
Maximum, Minimum	0, 19	0, 18	
Complications, n(%)	171(41.40)	173(41.89)	0.8877
Complications			0.5546
Mean (standard deviation)	0.8(1.30)	0.9(1.54)	
Median (Q1,Q3)	0.0(0.0, 1.0)	0.0(0.0, 1.0)	
Maximum, Minimum	0, 7	0, 9	
Number of complications per patients N (%)			0.4320
0	242(58.60)	240(58.11)	
1	87(21.07)	74(17.92)	
2	39(9.44)	41(9.93)	
≥3	45(10.90)	58(14.04)	
Clavien‒Dindo grading system, N (%)			0.7082
Missing	147(266)	155(258)	
I	60(40.82)	65(41.94)	
II	76(51.70)	82(52.90)	
IV-a	11(7.48)	8(5.16)	
Pneumonia, N (%)	93(22.52)	101(24.46)	0.5114
Respiratory failure, N (%)	1(0.24)	3(0.73)	0.6241
Mediastinal infection, N (%)	0(0.00)	1(0.24)	1.0000
Pneumoderm, N (%)	2(0.48)	5(1.21)	0.4511
Myocardial infarction, N (%)	0(0.00)	1(0.24)	1.0000
Arrhythmia, N (%)	17(4.12)	23(5.57)	0.3308
Urinary infection, N (%)	0(0.00)	1(0.24)	1.0000
Incision infection, N (%)	0(0.00)	4(0.97)	0.1241
Chylothorax, N (%)	2(0.48)	0(0.00)	0.4994
Recurrent laryngeal nerve injury, N (%)	45(10.90)	60(14.53)	0.117
Anastomotic leakage, N (%)	15(3.63)	17(4.12)	0.7184
Thoracic gastric fistula, N (%)	1(0.24)	0(0.00)	1.0000
Tracheopleural fistula, N (%)	0(0.00)	1(0.24)	1.0000
Bronchopleural fistula, N (%)	0(0.00)	1(0.24)	1.0000
Local recurrence, N%			0.2216
Yes	40(9.69)	51(12.35)	
No	373(90.31)	362(87.65)	
Distant metastasis, N%			0.2078
Yes	37(8.96)	48(11.62)	
No	376(91.04)	365(88.38)	


NAC, neoadjuvant chemotherapy; N, number; MIE, minimally invasive oesophagectomy; * Statistically significant (p<0.05).

## Abbreviations

NAC: neoadjuvant chemotherapy
ESCC: oesophagealesophageal squamous cell carcinoma
PSM: propensity score matching
OS: overall survival
DFS: disease-free survival
RCTs: randomized controlled trials
CTCAE: Common Terminology Criteria for Adverse Events
EUS: endoscopic ultrasound
CT: computed tomography
ECT: emission computed tomography
PET: Positron emission tomography
N: number
BMI: Body Mass Index
NA: Not Available
TNM: Tumor/Node/Metastasis
G1: Well differentiated
G2: Moderately differentiated
G3: Poorly differentiated
CMI: Circumferential Margin Involvement
ypTNM: pathological tumour/node/metastasis
Q: Quarter
AC: Adjuvant Therapy
FN: Febrile Neutropenia
DCR: Docetaxel Cisplatin Fluorouracil
TP: paclitaxel and cisplatin
DP: docetaxel and cisplatin
